# EMG-Based Simulation for Optimization of Human-in-the-Loop Control in Simple Robotic Walking Assistance

**DOI:** 10.3390/jsan14060113

**Published:** 2025-11-25

**Authors:** Arash Mohammadzadeh Gonabadi, Nathaniel H. Hunt, Farahnaz Fallahtafti

**Affiliations:** 1 Institute for Rehabilitation Science and Engineering, Madonna Rehabilitation Hospitals, Omaha, NE 68118, USA; 2 Department of Biomechanics and Center for Research in Human Movement Variability, University of Nebraska at Omaha, Omaha, NE 68182, USA

**Keywords:** electromyography, human-in-the-loop optimization, surrogate models, optimization algorithms, exoskeleton, walking assistance, waist tether, simple robots, simulation-based personalization

## Abstract

Exoskeletons offer promising solutions for enhancing human mobility; however, personalizing assistance parameters to optimize physiological outcomes remains challenging. Human-in-the-loop (HIL) optimization has emerged as an effective strategy for tailoring device control, often using electromyography (EMG) as a real-time proxy for metabolic cost. This study simulates HIL optimization using surrogate models built from the average root mean square of the muscles’ activations (EMG-RMS) derived from treadmill walking trials with a robotic waist tether. Nine surrogate models were evaluated for prediction accuracy, including gradient boosting (GB), random forest, support vector regression, and Gaussian process variants. Seven global optimization algorithms were compared based on convergence time, EMG-RMS at optimum, and efficiency metrics. GB achieved the highest predictive accuracy (1.57% RAEP). Among optimizers, the gravitational search algorithm (GSA) produced the lowest EMG-RMS value (0.17 normalized units) and the fastest convergence (0.32 s), while particle swarm optimization (PSO) achieved 0.36 EMG-RMS in 1.61 s. These findings demonstrate the value of EMG-based simulation frameworks in guiding algorithm selection for HIL optimization, ultimately reducing the experimental burden in developing personalized exoskeleton assistance strategies.

## 1. Introduction

Wearable robotic devices, such as exoskeletons, offer substantial benefits in augmenting human locomotion, particularly for individuals with mobility impairments. However, achieving optimal assistance requires personalization to individual biomechanics and preferences, as generic control parameters often lead to suboptimal performance [1–3]. Human-in-the-loop (HIL) optimization addresses this challenge by iteratively adjusting device parameters based on real-time physiological feedback, aiming to optimize metrics such as metabolic cost or muscle activation [2]. Traditional HIL implementations often rely on metabolic cost as the objective function; however, its measurement typically requires indirect calorimetry, prolonged walking trials, and specialized equipment, thereby limiting practicality in many settings [1,2].

Surface electromyography (EMG) offers a viable alternative, enabling non-invasive measurement of muscle activation and supporting faster feedback loops for optimization [4]. Previous studies have demonstrated the effectiveness of EMG in tuning exoskeleton controllers, for example, in swing-phase assistance, where EMG has served as a surrogate marker for user effort [4]. Expanding upon these findings, simulated HIL frameworks using probabilistic models of physiological landscapes provide a pre-experimental testbed to evaluate and compare optimization strategies without the burden of extensive pilot testing [1,2]. Such simulations map assistance parameters—such as peak timing and magnitude—to the average root mean square of the muscles’ activations (EMG-RMS), enabling robust algorithm comparisons in noisy, high-dimensional parameter spaces [2].

Recent advances in machine learning (ML) have further enabled the prediction of EMG signals using biomechanical inputs such as ground reaction forces (GRF), kinematics, and kinetics [5]. For instance, artificial neural networks (ANNs) have demonstrated high predictive accuracy (R > 0.90) in estimating lower-limb EMG signals and joint moments directly from GRF during walking and running [5]. Similarly, response surface models have been used to estimate EMG activation profiles from gait kinematics and kinetics across various walking speeds, showing consistent agreement with experimental muscle behavior [6]. Additional work has applied support vector machines and neural networks to estimate EMG patterns from joint angles and inertial measurement unit (IMU) data, enabling real-time gait analysis without requiring direct EMG acquisition [7–10]. In musculoskeletal simulations, methods like computed muscle control (CMC), by using optimization methods, have been employed to predict muscle forces and activations from motion capture and force plate data, providing detailed estimations of metabolic power throughout the gait cycle [11]. Echo state networks (ESNs) have also achieved high-fidelity full-body kinematics and kinetics predictions based on EMG inputs, supporting potential applications in real-time exoskeleton control [12]. Systematic reviews highlight the growing use of EMG and electroencephalogram (EEG) signals in predicting gait kinematics and kinetics, with ML models consistently improving lower-limb motion estimation accuracy [13,14]. These models reduce the reliance on invasive or prolonged testing procedures and support the development of surrogate landscapes for simulation-based HIL optimization.

**Figure 1.**
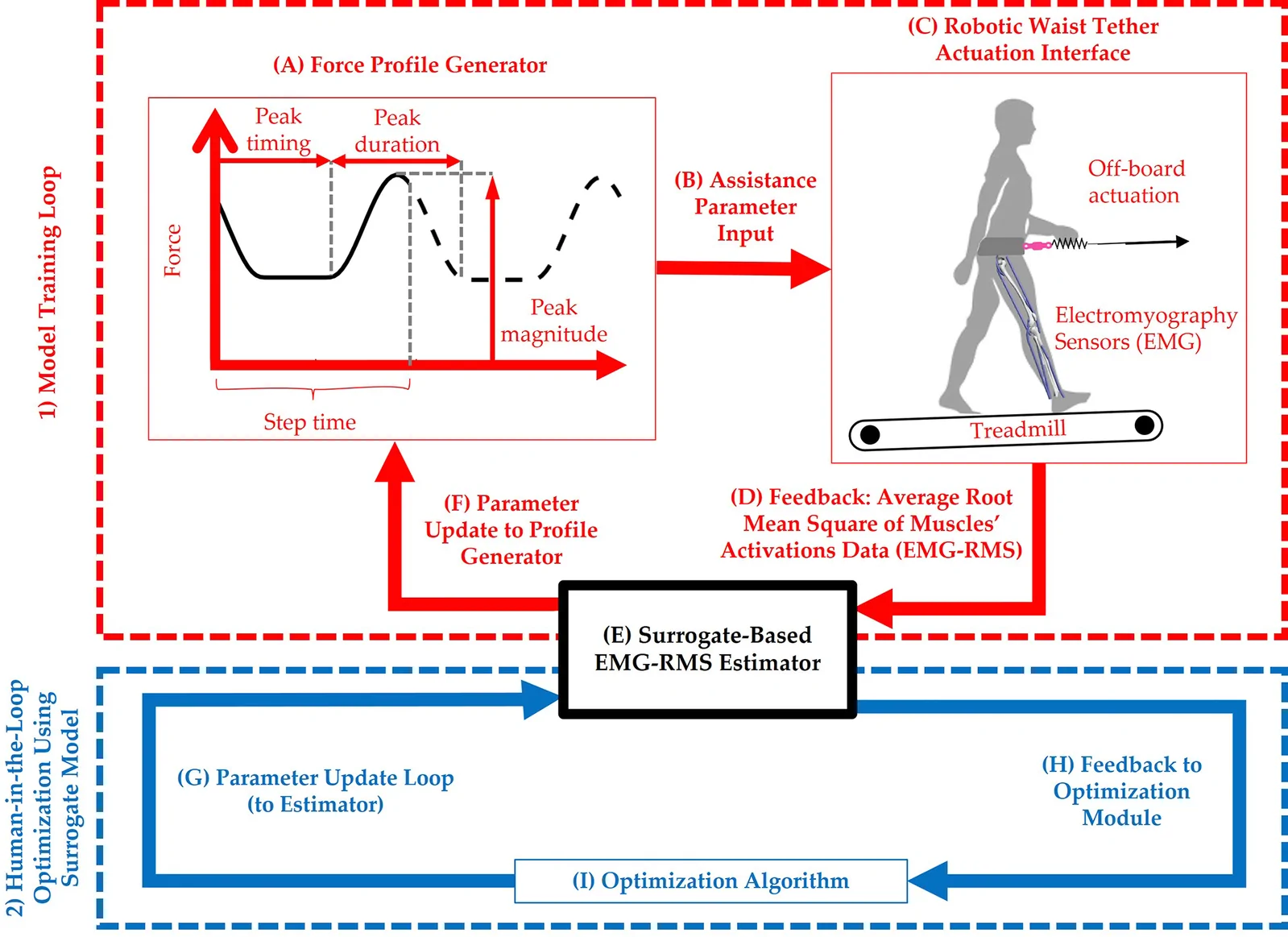
Overview of the human-in-the-loop framework for personalized robotic waist tether assistance. The system comprises two primary loops: **(1) Model Training Loop** (red): used to collect experimental data [21,22] and train a surrogate model for the root mean square of the muscles’ activations (EMG-RMS) prediction; and **(2) Human-in-the-Loop Optimization Loop Using Surrogate Model** (blue): employed to optimize assistance parameters based on predicted EMG-RMS (average root mean square of the muscles’ activations) values. (**A**) **Force Profile Generator** defines a piecewise semi-linear force profile parameterized by peak magnitude, peak timing, and peak duration. (**B**) **Assistance Parameter Input** transmits these parameters to the robotic waist tether controller. (**C**) **Robotic Waist Tether Actuation Interface** applies assistive force to the user via off-board actuation during treadmill walking. Seven surface EMG sensors were placed on the user’s right leg to measure muscle activity. Sensors were positioned over the Vastus Medialis, Tibialis Anterior, Soleus, Rectus Femoris, Gluteus Maximus, Gastrocnemius Medialis, and Biceps Femoris. (**D**) **Feedback (EMG-RMS):** EMG signals are processed to obtain the average RMS of muscle activations. (**E**) **Surrogate-Based EMG-RMS Estimator** uses a trained machine learning model to predict EMG-RMS based on assistance parameter input (timing, duration, magnitude). (**F**) **Parameter Update to Profile Generator** enables exploration of new assistance profiles during model training. (**G**) **Parameter Update Loop** passes proposed assistance parameters to the surrogate model. (**H**) **Feedback to Optimization Module** provides surrogate-predicted EMG-RMS values for each parameter set. (**I**) **Optimization Algorithm** selects and updates parameters iteratively to minimize predicted EMG-RMS. This framework enables efficient, data-driven tuning of robotic assistance strategies, reducing experimental workload while supporting individualized exoskeleton control.

Motivated by prior work that simulated metabolic cost landscapes to benchmark optimization strategies [1,2] and studies that employed EMG-informed Bayesian tuning for wearable assistance [4,15,16], the present study extends these frameworks to an EMG-based simulation context. Building on previously established computational pipelines [2], we aim to compare multiple surrogate models and optimization algorithms for identifying assistive parameter combinations that minimize EMG-RMS during treadmill walking with a robotic waist tether (see Figure 1). By integrating concepts from these foundational studies, this work contributes to simulation-based design and personalization of simple, wearable assistive systems.

Recent work has used muscle synergy–based HIL optimization to personalize hip-exoskeleton torque assistance, demonstrating the value of physiological structure (synergies) for real-time tuning [17]. Other studies have employed surface EMG as biofeedback to guide HIL trajectory selection for lower-limb exoskeleton motion planning, combining offline optimization with online human-guided refinement [18]. Building on these advances, the present study differs in three key ways. First, rather than solely using EMG as a control or feedback signal, we learn an EMG-RMS surrogate landscape over assistance parameters, enabling rapid, simulation-only exploration of parameter spaces before human trials. Second, we systematically compare nine surrogate models and seven global optimizers under a unified protocol, reporting accuracy, convergence efficiency, and sensitivity—information largely absent or implicit in synergy-based HIL and EMG-biofeedback HIL pipelines. Third, we formalize data integration and augmentation with explicit parameter justifications and sensitivity checks, creating a reproducible pre-screening tool that reduces experimental workload while maintaining physiological specificity. In contrast to metabolic-landscape simulations or our previous metabolic-optimization study, the present EMG-centric objective captures faster neuromuscular responses. It can thus better inform algorithm selection and initialization for subsequent human-in-the-loop experiments [9,15,17,18].

Complementary to earlier human-in-the-loop approaches that relied on EMG synergies or online biofeedback for exoskeleton tuning, the present framework provides a **simulation-based surrogate optimization environment** capable of evaluating algorithmic performance before human testing. This approach substantially reduces experimental time and participant burden by enabling pre-screening of optimizer behavior using EMG-RMS-based surrogate models derived from real physiological data. Moreover, by coupling machine-learning surrogates with global optimization and sensitivity analysis, the proposed framework bridges the gap between offline algorithm development and experimental HIL adaptation. As such, it offers a reproducible and generalizable methodology for identifying robust optimization strategies applicable across different assistive devices and user populations.

Building on our prior metabolic-based HIL simulation, the present study targets **EMG-RMS** to capture **rapid, muscle-specific** neuromuscular effort. Unlike metabolic rate—which requires long steady-state periods and reflects whole-body energetics—EMG provides **near-immediate feedback** at the level of **individual muscle groups**, aligning with muscle-engagement goals in rehabilitation and assistive control. This distinction is clinically meaningful for populations with **selective weakness, impaired endurance, or fatigue**, where short-bout HIL with muscle-targeted endpoints is preferred. Consequently, algorithm behavior and rank order observed under a metabolic objective may **not generalize** to EMG-driven optimization. Our framework, therefore, **re-benchmarks surrogate–optimizer pairs** for an EMG objective and **quantifies hyperparameter robustness**, providing actionable guidance for EMG-based HIL and laying groundwork for **dual-objective** studies (metabolic + EMG) in future experiments.

Exoskeleton personalization has advanced through HIL optimization and simulation targeting **metabolic cost, muscle synergy, and motion estimation**. However, most prior studies focused on individual joints (e.g., ankle or elbow), tested only a few optimizers, or relied solely on **real-time experiments**, limiting scalability and reproducibility. To address these gaps, this study presents a **simulation-based HIL framework** that benchmarks **nine machine-learning surrogates and seven global optimizers** for hip exoskeleton personalization. Using **EMG-RMS as a muscle-level proxy for metabolic effort**, the framework enables fast, physiologically relevant optimization while reducing experimental workload. Table 1 summarizes prior work and highlights this study as the **first comprehensive EMG-driven simulation framework** for pre-experimental hip exoskeleton optimization.

The primary research question addressed in this study is: How do different surrogate models and optimization algorithms perform in simulating EMG-based HIL optimization for exoskeleton walking assistance? Our objectives are: (1) to evaluate surrogate model accuracy in predicting EMG-RMS, and (2) to compare optimization algorithms on key performance metrics, including convergence time and solution quality. We hypothesized that Bayesian optimization (BO) would outperform heuristic approaches such as particle swarm optimization (PSO) in low-evaluation regimes, due to its probabilistic acquisition strategy that balances exploration and exploitation.

## 2. Materials and Methods

### 2.1. System Overview and Data Collection

This study simulated HIL optimization for a robotic waist tether system [21] to assist hip flexion during treadmill walking (Figure 1). The system architecture included a force profile generator parameterized by peak timing, duration, and magnitude; an assistance parameter input module; a feedback loop that computes average EMG-RMS from muscle activation data; a surrogate-based EMG-RMS estimator; a parameter update loop; and an optimization algorithm module.

EMG data were obtained from lower-limb muscles (i.e., vastus medialis, tibialis anterior, soleus, rectus femoris, gluteus maximus, gastrocnemius medialis, and biceps femoris muscles) during treadmill walking trials [21,22] and processed to yield normalized RMS values, which served as the objective function for optimization. Specifically, kinematic and kinetic data were sourced from a publicly available dataset that investigated gait under various footwear conditions and perturbations [21,22]. The dataset included motion capture recorded at 120 Hz and GRF measured at 1000 Hz from 10 healthy participants (age: 28.0 ± 4.7 years, body mass: 83.2 ± 12.2 kg, height: 1.80 ± 0.05 m, leg length: 0.993 ± 0.036 m; mean ± SD) walking at a fixed speed of 1.25 m/s. Perturbations were applied through a robotic waist tether, enabling simulation of the effects of exoskeleton assistance on gait mechanics. In the experiment reported in [21,22], participants walked on a treadmill while wearing shoes with varying inclinations to induce mechanical perturbations, allowing for detailed analysis of GRF, joint angles, and joint moments [21,22].

EMG recordings were supplemented using another publicly available dataset focused on perturbation-based metabolic cost estimation [22]. This dataset provided surface EMG signals from key lower-limb muscles (i.e., vastus medialis, tibialis anterior, soleus, rectus femoris, gluteus maximus, gastrocnemius medialis, and biceps femoris muscles) during treadmill walking with force perturbations applied at the center of mass via a robotic waist tether [21,22]. Participants walked under 36 distinct force profiles (36 conditions) presented in a randomized order and divided into three blocks, each separated by a 10 min rest period. Thirty-two of these conditions involved sinusoidal force profiles, created by varying three parameters: peak duration (33%, 66%, and 99% of step time), desired peak timing (0%, 25%, 50%, and 75% of step time), and target peak net aiding force magnitudes (ranging from 4% to 24% of body weight). All force values are reported as net aiding forces; to account for the treadmill’s incline, the actual tether forces applied were 5% higher to offset the additional gravitational component. EMG was collected to assess neuromuscular responses and estimate associated metabolic changes [21,22].

To ensure compatibility between datasets, signals were preprocessed to align sampling rates: EMG and GRF were interpolated and resampled to 1000 Hz, while motion capture data remained at 120 Hz. All signals were time-normalized to 100 points per gait cycle to enable consistent trial comparisons [21,22].

The objective was the average root mean square (RMS) of the muscle activation signals, computed over a complete gait cycle and across seven muscles (Vastus Medialis, Tibialis Anterior, Soleus, Rectus Femoris, Gluteus Maximus, Gastrocnemius Medialis, and Biceps Femoris) on the right leg. EMG signals were recorded at 2000 Hz and processed following established guidelines [21,22]. The raw signals were band-pass filtered (20–450 Hz), rectified, and low-pass filtered using a second-order Butterworth filter with a 6 Hz cutoff to create the linear envelope [21,22]. RMS was calculated using a 300 ms moving window with 50% overlap [21,22]. All EMG signals were normalized to baseline (no-exoskeleton) walking trials and expressed in dimensionless units. The final cost function was computed as the mean of normalized RMS values across all seven muscles and strides for each trial [1,2]. Inputs to the surrogate model included three biomechanical assistance parameters: torque magnitude (Nm/kg), onset timing (gait cycle %), and duration (gait cycle %).

It is important to note that the datasets referenced in [21,22] are complementary data releases from the same experimental study, not separate or independent experiments. The earlier publication by Antonellis et al. [21] detailed the experimental protocol and perturbation conditions. It released the kinematic and kinetic recordings, while the later dataset by Dzewaltowski et al. [22] reported the same participant cohort and identical experimental setup, providing the surface EMG recordings that were not included in the initial release. Both datasets involved the same ten healthy young adults walking at 1.25 m/s under identical waist-tether perturbations using the same instrumentation and data-processing pipeline. This integration, therefore, reconstructs the full multimodal dataset from a single experiment rather than merging heterogeneous sources, ensuring full compatibility and preventing demographic or methodological bias in model training. EMG signals were recorded from seven lower-limb muscles at 2000 Hz and processed by band-pass filtering (20–450 Hz), rectification, and low-pass filtering (6 Hz cutoff, 2nd-order Butterworth) to create linear envelopes [21–23]. RMS values were computed using a 300 ms window with 50% overlap and normalized to baseline Zero-Force walking [21,22]. Each trial included ~50 strides per condition, producing ~18,000 strides in total across all participants [21,22]. Sampling durations (~1.8 h per session) and sensor synchronization matched those reported in the original publications, ensuring data compatibility and minimizing potential biases between datasets.

Surrogate models [1,2] were constructed to approximate the EMG-RMS landscape as a function of normalized assistance parameters (peak timing, peak magnitude, and peak duration). The combined dataset included 360 valid experimental trials (36 conditions × 10 participants) along with 200 synthetic trials [1,2]. This data augmentation improved the surrogate model’s generalization ability across various assistance profiles [1,2]. The evaluated models included linear ridge (LR) regression, polynomial ridge (PR) regression, Gaussian process (GP) models with various kernels—squared exponential (GPSE), Matérn 3/2 (GPM), absolute exponential (GPAE), and rational quadratic (GPRQ)—as well as support vector regression (SVR), random forest (RF), and gradient boosting (GB) [1,2] (Table 2). Model performance was assessed using the relative absolute error between predicted and actual EMG-RMS values [1,2].

### 2.2. Model Development and Optimization Setup

To enhance model robustness, data augmentation techniques were employed, including the addition of Gaussian noise (σ = 0.01–0.05) [1,2] and polynomial interpolation to generate synthetic samples within the assistance parameter space [1,2]. This process expanded the dataset by approximately 50%, helping to mitigate overfitting in sparsely sampled regions [1,2]. Each model was trained using five-fold cross-validation (k = 5) repeated across 200 iterations, yielding 1000 (5 × 200) randomized splits [1,2]. Hyperparameter tuning was conducted using grid search combined with 5-fold cross-validation [1,2].

Tuned parameters included regularization strength (λ = 0.1–10) for LR and PR models, kernel bandwidth (σ = 0.1–1) for GP variants, and tree depth (range: 3–10) for RF and GB models [1,2]. The dataset was split into training (80%) and testing (20%) subsets [1,2]. Seven global optimization algorithms [1,2] were implemented to minimize surrogate-predicted EMG-RMS: Bayesian Optimization (BO), Exploitative Bayesian Optimization (EBO), Genetic Algorithm (GA), Particle Swarm Optimization (PSO), Covariance Matrix Adaptation Evolution Strategy (CMAES), Cross-Entropy (CE), and Gravitational Search Algorithm (GSA) (Table 2). Each algorithm was run for 100 evaluations [1,2]. Performance was assessed using four metrics: mean time to convergence (in seconds) [1,2], EMG-RMS at the optimum (normalized units) [1,2], area under the convergence curve (AUC) [1,2], and average rate of improvement (ARI, scaled × 10^−5^) [1,2].

To account for human adaptation effects, simulations incorporated stochastic noise models representing inter-individual variability, following prior approaches [1,2]. Convergence was defined as achieving less than a 1% change in the objective function over the final 10 iterations [1,2]. Each optimization algorithm was executed across 10 independent trials (re-running 10 times) to ensure robustness against stochastic variability in initialization and search trajectory, with 100 evaluations per trial (a total of 10 × 100 evaluations) to reflect a realistic limit on experimental feasibility in HIL studies [1,2]. Gaussian noise (σ = 0.046) was added to surrogate predictions to simulate realistic variability, reflecting typical experimental measurement noise [1,2]. Each algorithm was initialized with a uniform normalized distribution across the parameter space (peak magnitude, peak timing, and peak duration) [1,2], ranging from 0 to 1. All simulations and analyses were conducted using MATLAB R2024b (MathWorks Inc., Natick, MA, USA), employing built-in functions and custom scripts consistent with prior surrogate modeling and optimization workflows [1,2].

To ensure a fair comparison across all optimization methods, each algorithm operated within normalized bounds [0, 1] for Peak Magnitude, Peak Timing, and Peak Duration. The maximum number of function evaluations was set to 100 per run for all methods. The configuration parameters, including population size, initialization strategy, exploration constant, and iteration limits, are summarized in Table 3. These initial values were inspired by established simulation studies in the literature [1,2,5]. Random initialization was controlled using a fixed random seed (rng(42)) to ensure reproducibility.

**Figure 2.**
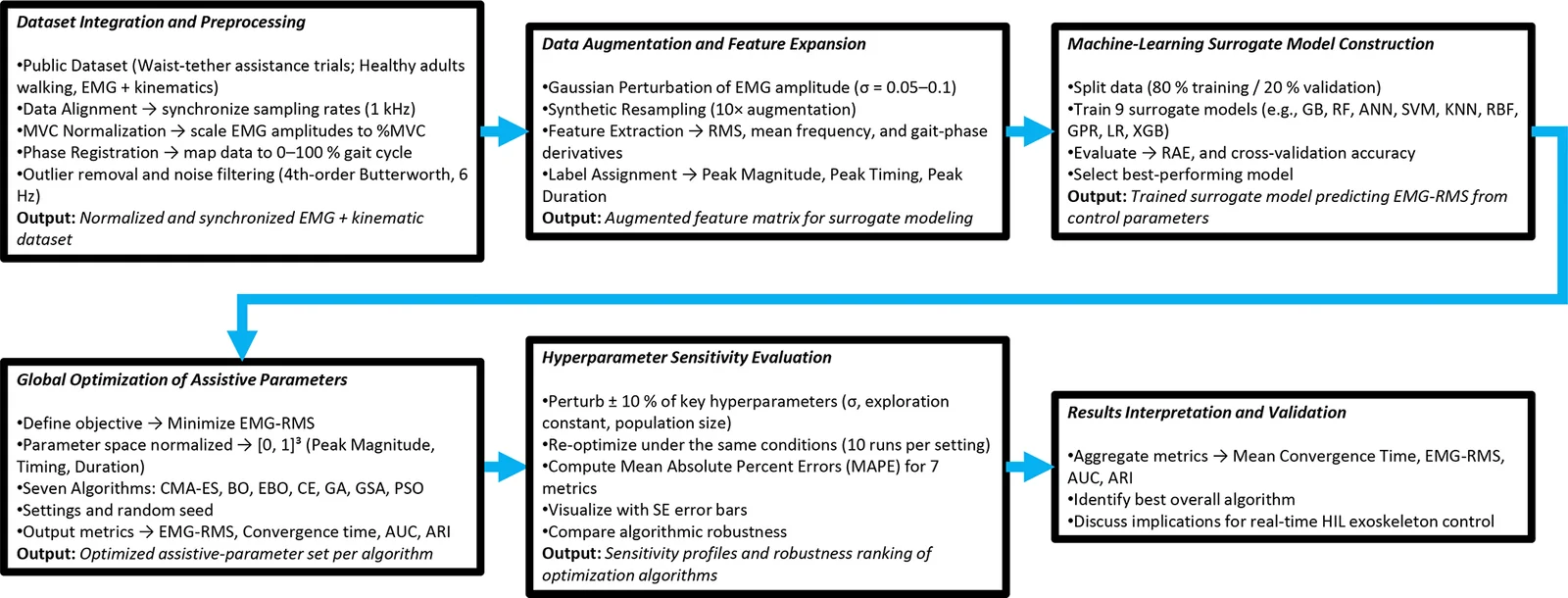
Overall workflow of the EMG-based simulation and optimization framework. The schematic illustrates the entire process—from raw data collection to performance evaluation—showing dataset integration, augmentation, surrogate modeling, optimization, and sensitivity analysis steps used in the study. The arrows indicate the direction of progression from one step to the next.

The overall process used in this study is summarized in Figure 2. The workflow begins with integrating public EMG and kinematic datasets, followed by signal preprocessing (resampling to 1000 Hz, amplitude normalization to MVC, and phase registration to 0–100% gait cycle). Augmentation is then applied through Gaussian perturbation (σ = 0.05–0.1) and random temporal shifts (±5%), producing tenfold expanded datasets for model training. The next stage involves training surrogate models to predict EMG-RMS responses from assistive parameters (Peak Magnitude, Peak Timing, Peak Duration). The surrogate predictions are then used in a global optimization pipeline to minimize EMG-RMS under seven optimization algorithms. Finally, the framework includes a sensitivity analysis step to evaluate the robustness of each optimizer to small hyperparameter changes.

The optimization objective was the **normalized EMG-RMS** averaged over the seven instrumented muscles during steady treadmill walking. EMG-RMS was selected to enable **rapid, muscle-specific feedback** and to support **short-duration HIL protocols**. We anticipated differences in search dynamics and optimizer rankings because EMG landscapes can exhibit sharper local structure and task- or muscle-dependent curvature compared with metabolic landscapes [2]. We therefore re-evaluated seven global optimizers under identical budgets and added a **hyperparameter sensitivity analysis** (±10% changes in initial σ, exploration constant, population/agent size, or swarm size) to assess **robustness** of convergence time, assistance parameters (Peak Magnitude, Peak Timing, Peak Duration), and outcome metrics (EMG-RMS, AUC, ARI). Algorithm configurations are summarized in Table 3, with initial values **inspired by prior HIL optimization literature** to ensure comparability and reproducibility.

To formalize the EMG-based surrogate estimation and optimization procedure, the relationship between assistive parameters and neuromuscular response can be expressed as follows:

(1) $$x=[x_{1},x_{2},x_{3}]=[Mpeak,Tpeak,Dpeak]$$

where x is the vector of normalized assistive parameters: $x_{1}$ is *Mpeak* (peak magnitude of the assistive force), $x_{2}$ is *Tpeak* (timing of the peak force within the gait cycle), and $x_{3}$ is *Dpeak* (duration of the assistive profile as a percentage of the gait cycle). All parameters are normalized within the range [0, 1 ] for comparability across algorithms. The true but unknown mapping between assistive parameters and the resulting EMG-RMS response can be described as:

(2) y=f(x)+ε

where y denotes the observed mean EMG-RMS value aggregated across the seven instrumented muscles, f(x) is the underlying nonlinear function representing the physiological relationship between assistive parameters and EMG response, and ε represents measurement noise or unmodeled variability.

The surrogate model $\hat{f}(x;\theta )$, parameterized by θ, is trained using the experimental data ${\left\{(x_{i},y_{i})\right\}}_{i=1}^{N}$ to minimize the mean squared prediction error:

(3) $$\theta^{*}=\arg\;\underset{\theta }{\min}\frac{1}{2}\sum_{i=1}^{N}[y_{i}-\hat{f}(x_{i};\theta ){]}^{2}$$

where N is the total number of samples, $y_{i}$ is the measured EMG-RMS for the $i^{th}$ trial, and $\hat{f}(x;\theta )$ is the surrogate model’s predicted EMG-RMS for the same assistive parameter input. The optimized parameters $\theta^{*}$ represent the best-fitting model configuration for EMG prediction.

Once trained, the surrogate model is used as an objective function for global optimization:

(4) $$x^{*}=\arg\;\underset{x\in {\left[0,1\right]}^{3}}{\min}\hat{f}(x;\theta^{*})$$

where $x^{*}$ denotes the **optimal combination of assistive parameters** that minimizes the predicted EMG-RMS response. The optimization algorithms (CMA-ES, BO, EBO, CE, GA, GSA, and PSO) iteratively update x to approach this minimum, identifying the most efficient assistance settings. This formulation defines a unified **surrogate-based optimization framework**, in which the surrogate model approximates the nonlinear EMG landscape, and the optimizer searches this landscape to determine the assistive parameters that minimize overall muscle activation effort.

## 3. Results

**Figure 3.**
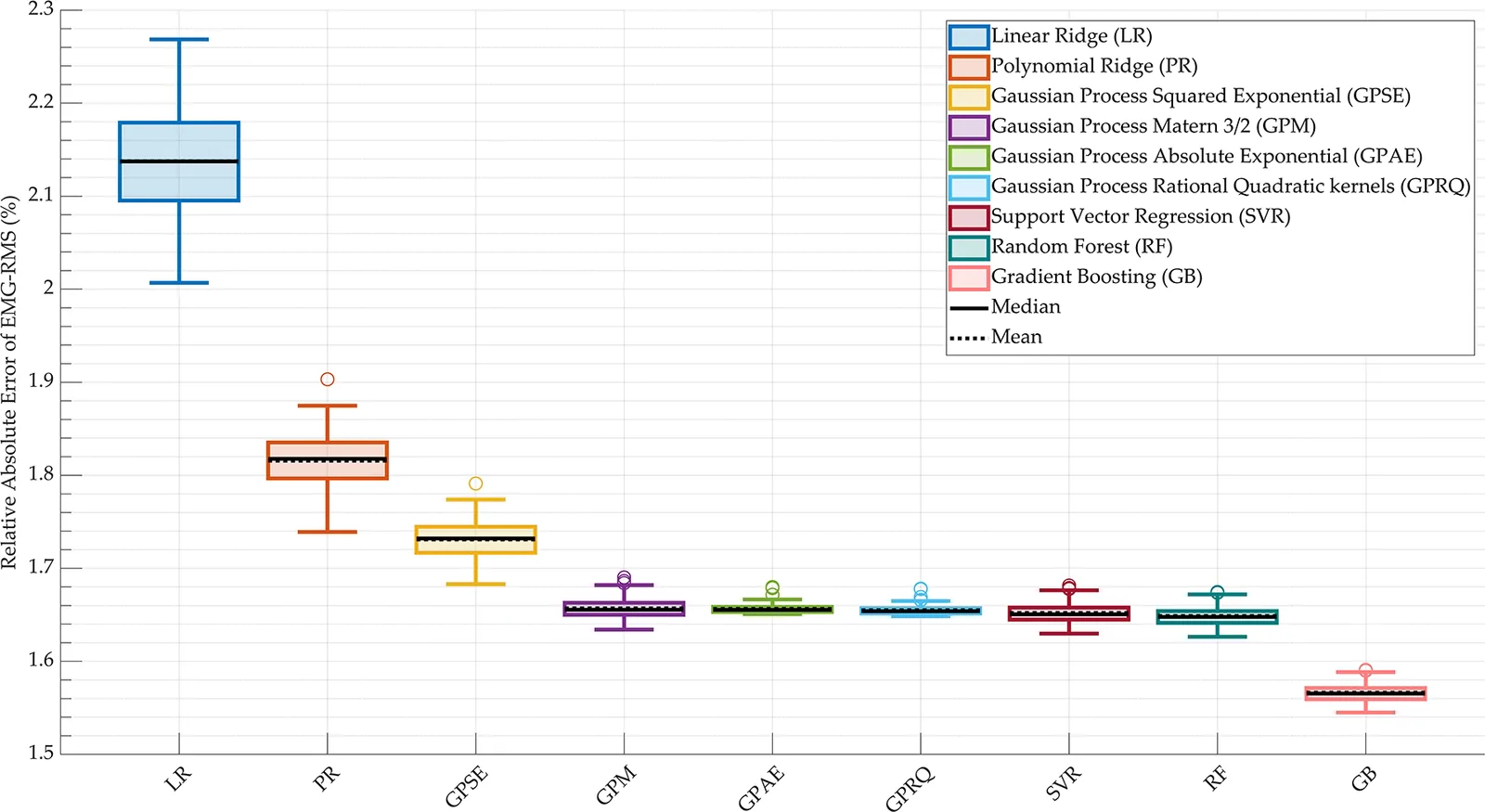
Comparison of surrogate model performance based on relative absolute error percentage (RAEP) of the root mean square of the muscles’ activations (EMG-RMS) predictions. Boxplots illustrate RAEP distributions across 200 iterations of 5-fold cross-validation for nine machine learning models, arranged from highest to lowest mean RAEP (left to right): linear ridge (LR), polynomial ridge (PR), four Gaussian process (GP) variants—squared exponential (GPSE), Matérn 3/2 (GPM), exponential (GPAE), and rational quadratic (GPRQ)—support vector regression (SVR), random forest (RF), and gradient boosting (GB). In each boxplot, the central line indicates the median RAEP value, the box denotes the interquartile range, and the dotted line overlays the mean RAEP across models. The predicted variable is the normalized average RMS computed from seven surface EMG sensors placed on the participant’s right leg, targeting the vastus medialis, tibialis anterior, soleus, rectus femoris, gluteus maximus, gastrocnemius medialis, and biceps femoris muscles during treadmill walking. The circles in the boxplots represent outlier data points that fall outside 1.5 times the interquartile range.

The evaluation of surrogate models identified GB as the top-performing approach, achieving the lowest RAE of 1.57%, followed by RF at 1.65%, and GP variants ranging from 1.65% to 1.73%. Linear models—LR: 2.14% and PR: 1.82%—exhibited higher error rates, indicating limited capacity to model the nonlinear structure of EMG-RMS landscapes (Figure 3; Table 4). Prediction accuracy remained consistent across cross-validation folds, with GB demonstrating superior generalization. Data augmentation effectively reduced overfitting, and adding Gaussian noise improved test performance in GP models. A summary of model performance metrics is presented in Table 4.

**Figure 4.**
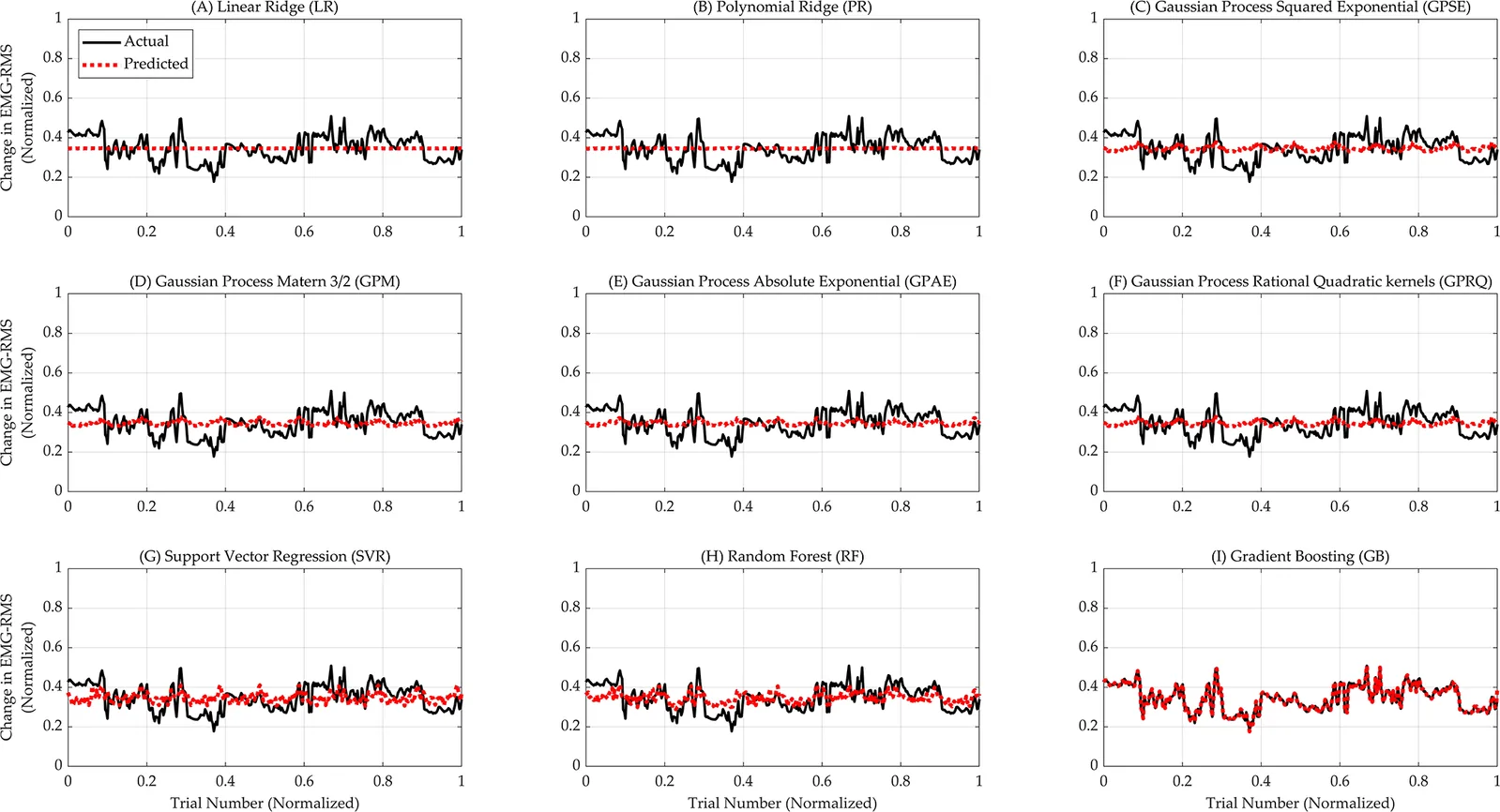
Trial-wise comparison of actual versus predicted average of the root mean square of the muscles’ activations (EMG-RMS) across all surrogate models. Each subplot displays predicted (red dashed line) and actual (black line) normalized EMG-RMS values for 100 test trials of a single model. The x-axis represents trial indices normalized to the [0, 1] range to facilitate pattern alignment and cross-model comparison rather than raw trial ordering. Subplots correspond to: (**A**) linear ridge (LR), (**B**) polynomial ridge (PR), (**C**) Gaussian process with squared exponential kernel (GPSE), (**D**) Gaussian process with Matérn 3/2 kernel (GPM), (**E**) Gaussian process with absolute exponential kernel (GPAE), (**F**) Gaussian process with rational quadratic kernel (GPRQ), (**G**) support vector regression (SVR), (**H**) random forest (RF), and (**I**) gradient boosting (GB). These plots highlight GB’s superior predictive performance and consistency at the trial level for EMG-RMS estimation. EMG signals were recorded using seven surface electrodes placed on the user’s right leg, targeting the Vastus Medialis, Tibialis Anterior, Soleus, Rectus Femoris, Gluteus Maximus, Gastrocnemius Medialis, and Biceps Femoris muscles during treadmill walking.

**Figure 5.**
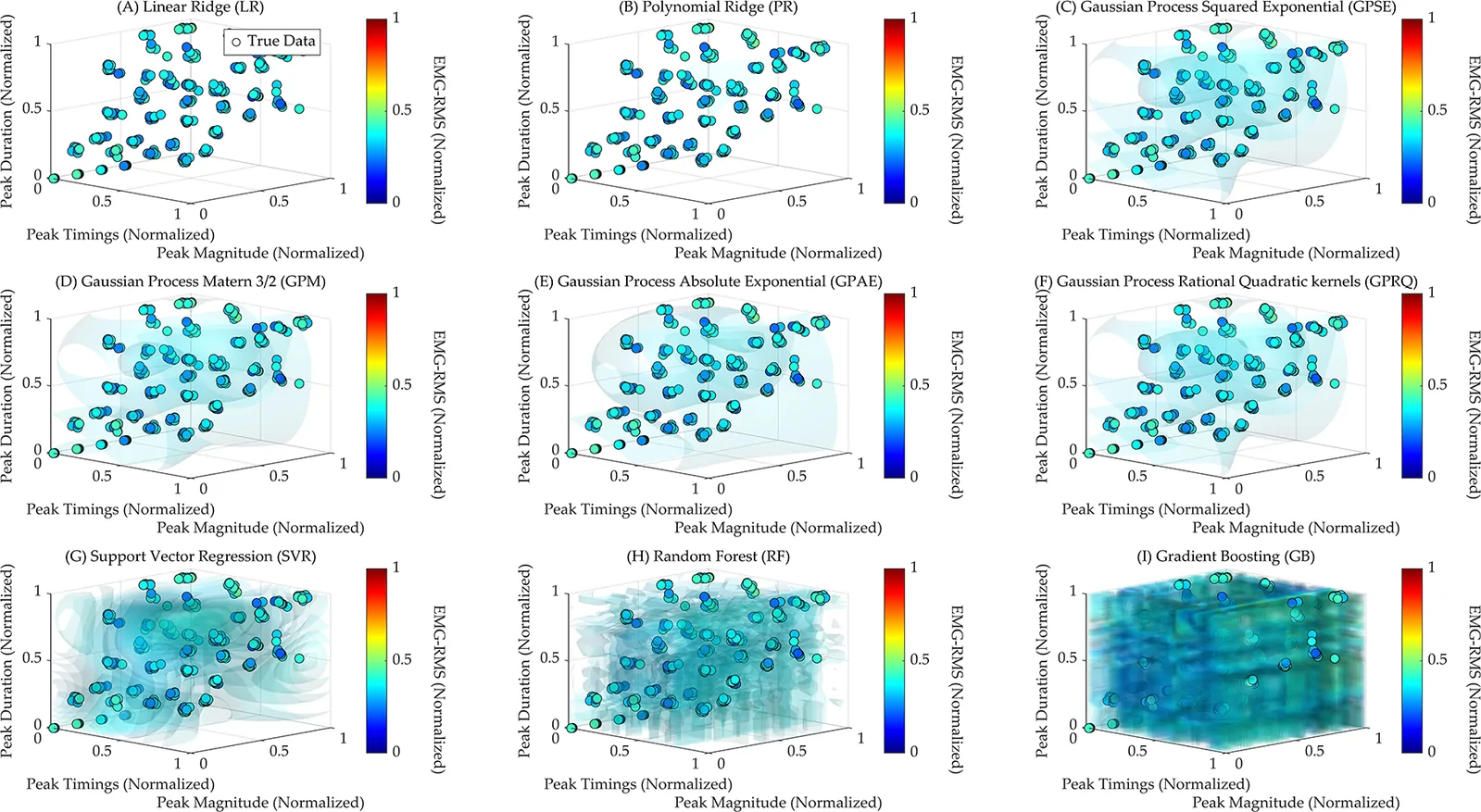
Predicted average root mean square of the muscles’ activations (EMG-RMS) landscapes with overlaid experimental data points for each surrogate model. Filled contour plots depict predicted normalized EMG-RMS across the assistance parameter space, defined by peak timing (x-axis), peak magnitude (y-axis), and peak duration (z-axis). Overlaid circles represent actual data points used for model training. Subplots correspond to: (**A**) linear ridge (LR), (**B**) polynomial ridge (PR), (**C**) Gaussian process with squared exponential kernel (GPSE), (**D**) Gaussian process with Matérn 3/2 kernel (GPM), (**E**) Gaussian process with absolute exponential kernel (GPAE), (**F**) Gaussian process with rational quadratic kernel (GPRQ), (**G**) support vector regression (SVR), (**H**) random forest (RF), and (**I**) gradient boosting (GB). These visualizations highlight GB’s superior spatial expressiveness and alignment with the experimental data distribution. EMG signals were recorded using seven surface electrodes placed on the user’s right leg, targeting the Vastus Medialis, Tibialis Anterior, Soleus, Rectus Femoris, Gluteus Maximus, Gastrocnemius Medialis, and Biceps Femoris muscles during treadmill walking.

Predicted versus actual EMG-RMS values were plotted for each surrogate model to assess accuracy (Figure 4), and three-dimensional surrogate landscapes were generated to visualize optimal regions within the parameter space (Figure 5).

Landscape visualizations (Figure 5) confirmed that GB produced the most detailed and expressive topography, with sharp gradients capturing nonlinear interactions among assistance parameters. Overlaid experimental data points strongly aligned with GB and RF predictions, further validating their spatial accuracy and ability to generalize across the parameter space (Figure 4).

A summary of the optimization results is presented in Table 5. Optimization results revealed the GSA as the most effective method, converging to the almost lowest EMG-RMS value (0.17 normalized units) in just 0.32 s, with an AUC of 0.12 and an ARI of 2.84 × 10^−5^ (Table 5). The PSO closely followed, achieving an optimum of 0.36 in 1.61 s, with an AUC of 0.10 and an ARI of 0.62 × 10^−5^, demonstrating strong performance in rapid exploration of the parameter space (Table 5). BO and EBO showed moderate performance, converging to optima between 0.16 and 0.20, with 8.74 to 9.90 s of computational time. Although CMAES, CE, and GA achieved relatively fast run times (<1.7 s), they underperformed in minimizing EMG-RMS.

**Figure 6.**
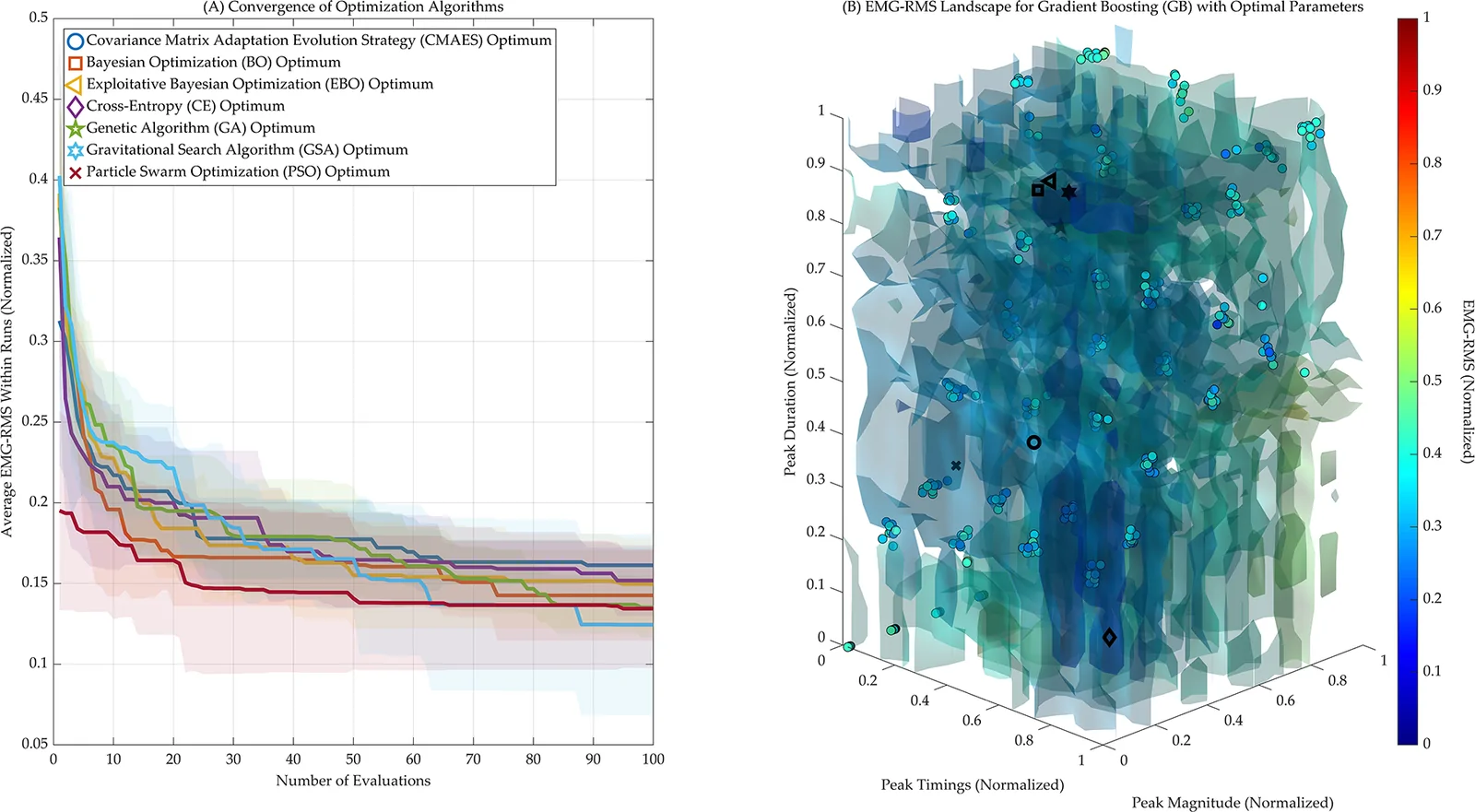
Optimization convergence and parameter landscape visualization. (**A**) Normalized convergence trajectories of the average root mean square of the muscles’ activations (EMG-RMS) across 100 iterations within each run, as the gradient boosting (GB) surrogate model predicted. The evaluated algorithms include Covariance Matrix Adaptation Evolution Strategy (CMAES), Bayesian Optimization (BO), Exploitative Bayesian Optimization (EBO), Cross-Entropy (CE), Genetic Algorithm (GA), Gravitational Search Algorithm (GSA), and Particle Swarm Optimization (PSO). (**B**) The predicted EMG-RMS landscape generated by the GB model is visualized as a filled 3D contour plot over the assistance parameter space, which is defined by peak magnitude, peak timing, and peak duration. Final optimal solutions identified by each algorithm are overlaid, showing convergence within a low-cost region. EMG signals were recorded using seven surface electrodes placed on the user’s right leg, targeting the Vastus Medialis, Tibialis Anterior, Soleus, Rectus Femoris, Gluteus Maximus, Gastrocnemius Medialis, and Biceps Femoris muscles during treadmill walking. The combined legend explains the colors and shapes used across all subplots, reducing visual clutter and making the figure easier to interpret.

Convergence trajectories for all optimization algorithms were displayed (Figure 6), with an exemplar GB-generated landscape overlaid in Figure 6B.

**Figure 7.**
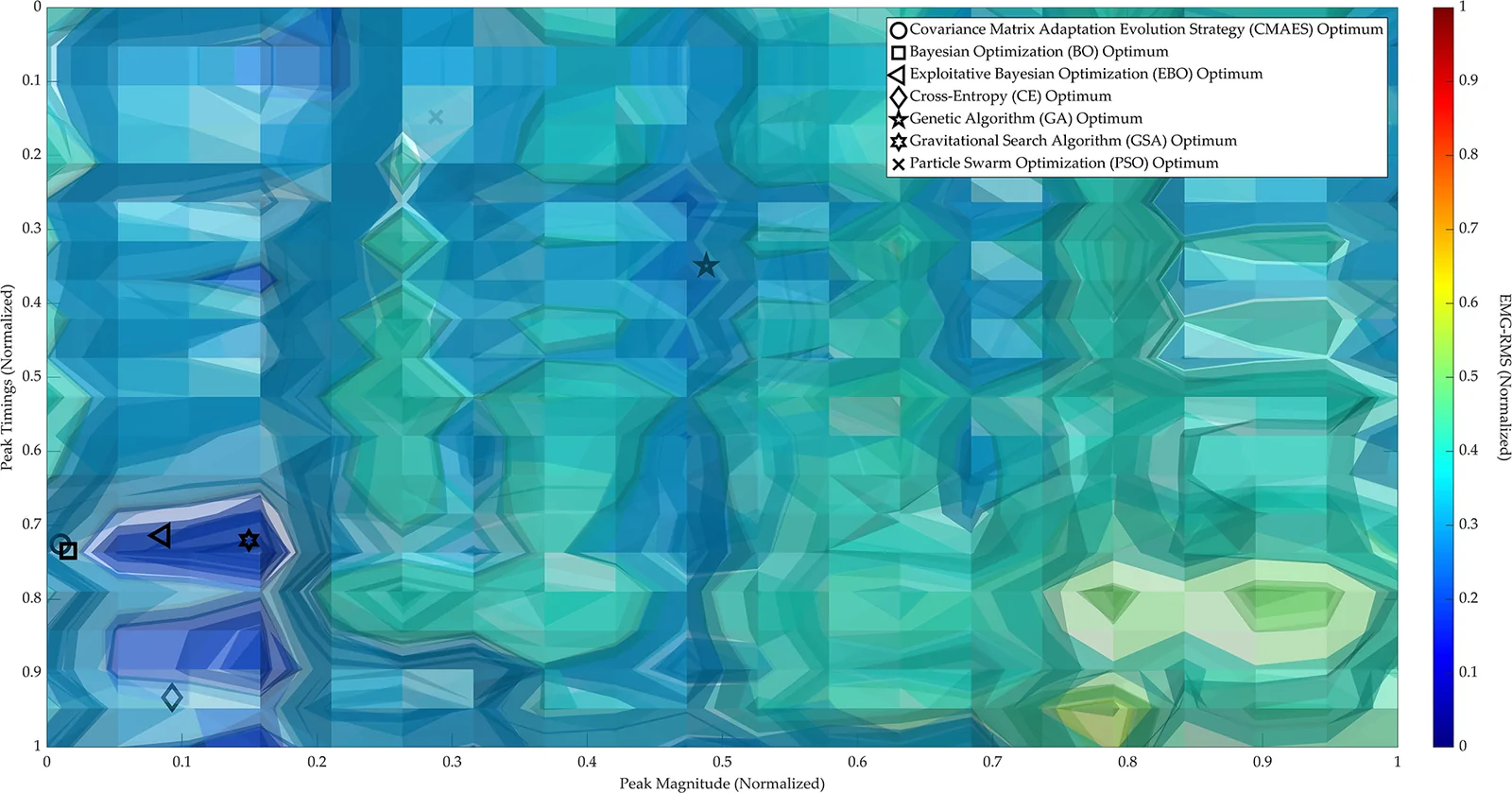
Optimization convergence and parameter landscape visualization. The top-view (two cross sections of peak timing–magnitude plane at the highest peak duration) filled 3D contour visualization of the average root mean square of the muscles’ activations (EMG-RMS) cost landscape predicted by the gradient boosting (GB) model, shown across the assistance parameter space (peak magnitude, peak timing, and peak duration). The figure highlights optimal solutions identified by each optimization algorithm, demonstrating convergence patterns within the low-cost region. A lighter color density scheme is used to visualize better clustering of solutions and the smooth valleys that facilitate efficient search, particularly by ensemble-based surrogates like GB. This visualization underscores the presence of shallow, navigable cost valleys that support steady algorithmic progress. EMG signals used in model training were derived from seven surface electrodes placed on the participant’s right leg (Vastus Medialis, Tibialis Anterior, Soleus, Rectus Femoris, Gluteus Maximus, Gastrocnemius Medialis, and Biceps Femoris) during treadmill walking.

Figure 7 provides a top-view visualization of the GB-predicted EMG-RMS cost landscape across the timing–magnitude plane at the highest peak duration, revealing a smooth, low-cost valley structure. Most optimization algorithms converged to clustered optima within this region, highlighting the surrogate’s expressiveness and the efficiency of global search methods in navigating the parameter space.

**Figure 8.**
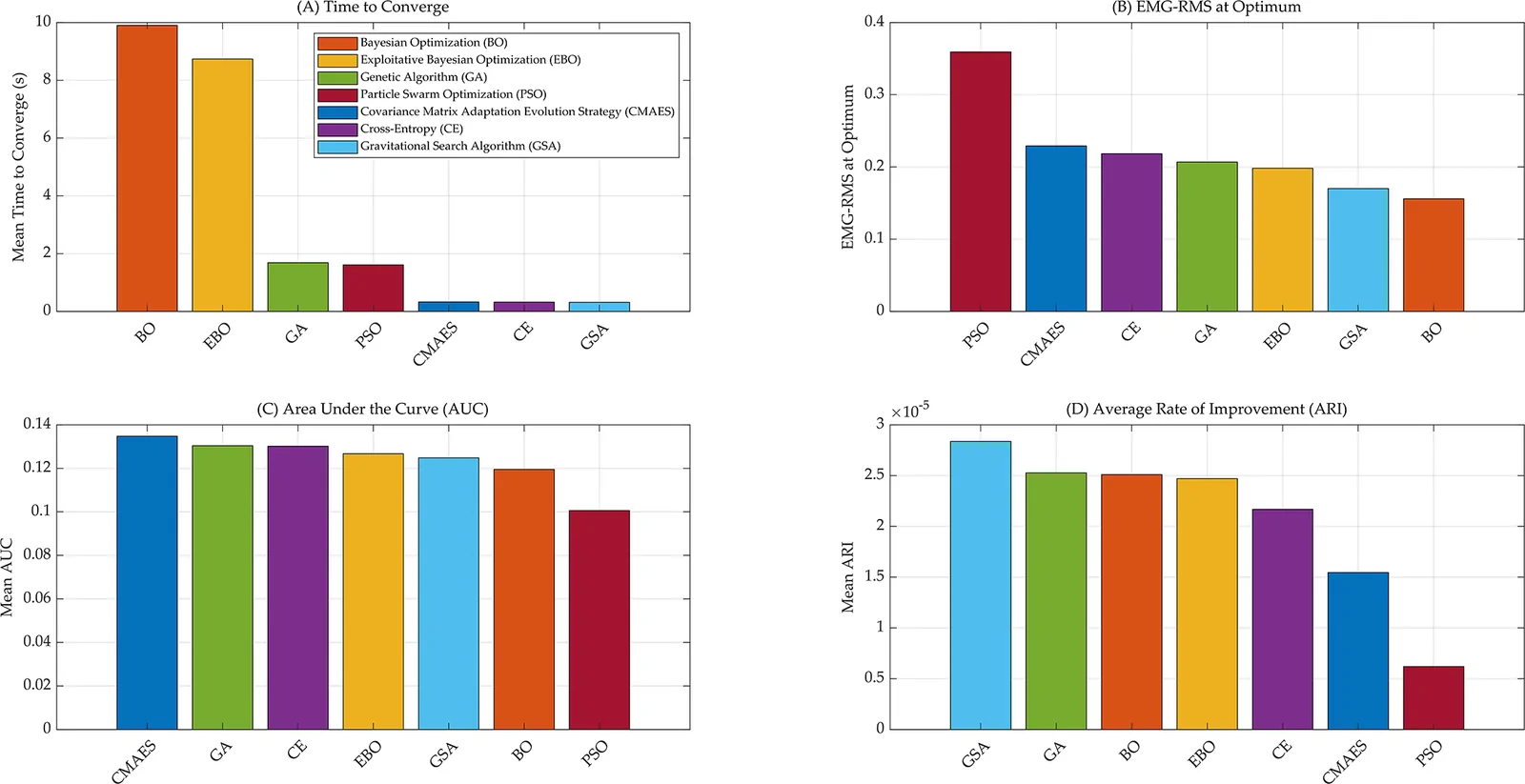
Optimization algorithm performance comparison. Comparative performance metrics of seven global optimization algorithms: Covariance Matrix Adaptation Evolution Strategy (CMAES), Bayesian Optimization (BO), Exploitative Bayesian Optimization (EBO), Cross-Entropy (CE), Genetic Algorithm (GA), Gravitational Search Algorithm (GSA), and Particle Swarm Optimization (PSO). (**A**) The mean time to convergence illustrates BO and EBO’s higher computational cost than the other methods. (**B**) Each algorithm achieved the final normalized average of the root mean square of the muscles’ activations (EMG-RMS), with GSA yielding the lowest value. (**C**) Mean area under the convergence curve (AUC), where lower values indicate greater optimization efficiency. (**D**) The average rate of improvement (ARI) represents the per-iteration rate of cost reduction, with EBO exhibiting the steepest improvement trajectory. EMG signals were recorded using seven surface electrodes placed on the user’s right leg, targeting the Vastus Medialis, Tibialis Anterior, Soleus, Rectus Femoris, Gluteus Maximus, Gastrocnemius Medialis, and Biceps Femoris muscles during treadmill walking.

Convergence curves (Figure 6A) illustrated GSA’s efficiency in navigating multimodal landscapes, with most optimal solutions clustering around a peak magnitude of approximately 0.15 Nm/kg, a peak timing of ~0.72, and a peak duration of ~0.97 of the gait cycle (Figure 6B). Across repeated trials, optimization results were consistent, with intertrial variability in the identified optima remaining below 5%, supporting the framework’s robustness. A summary of the optimization algorithm performance is presented in Figure 8.

**Figure 9.**
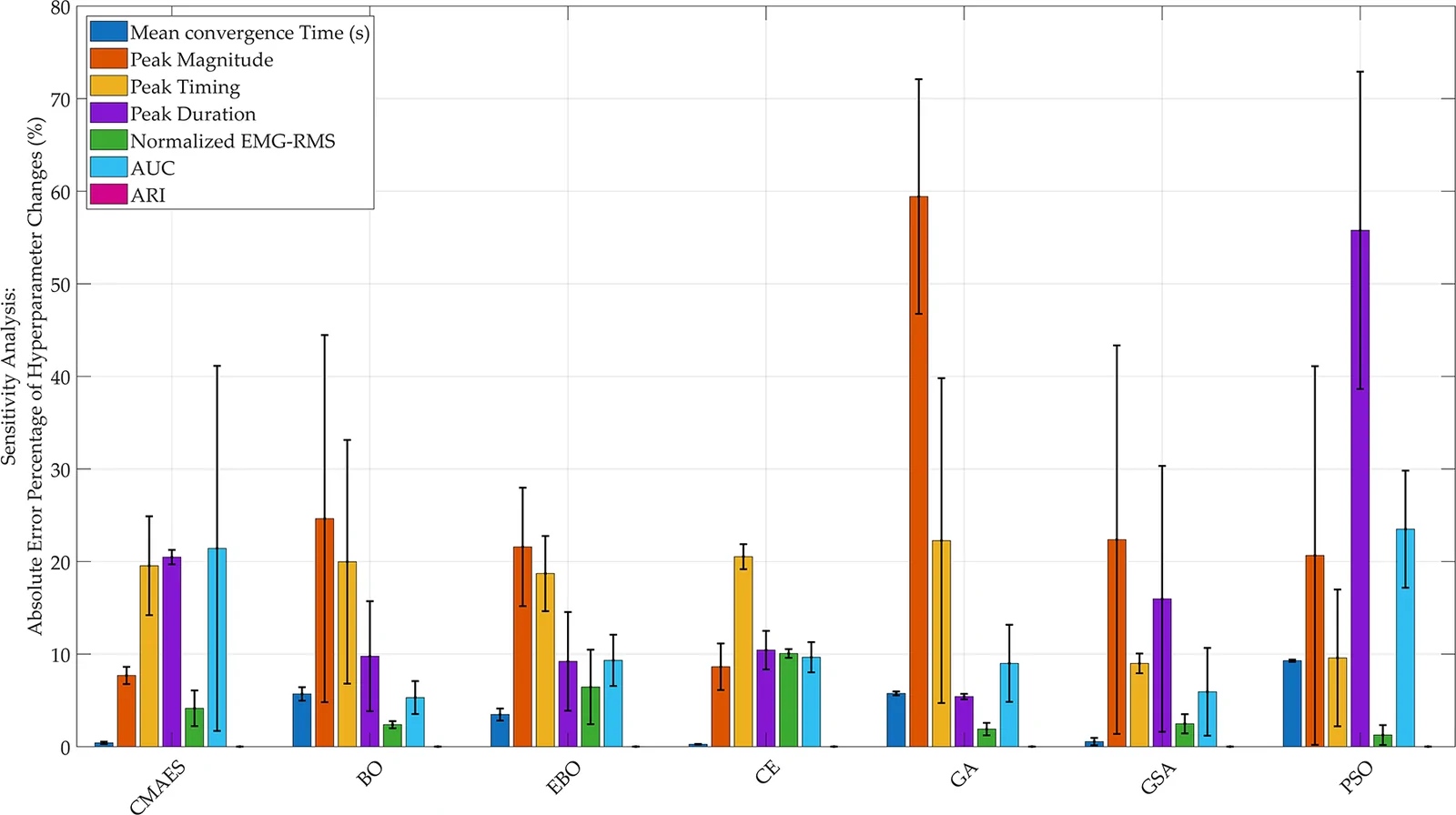
Sensitivity analysis of optimization algorithms for exoskeleton assistance parameters. The bar plot displays the mean absolute percent errors (relative to nominal 0% hyperparameter change) for −10% and +10% changes in initial σ, exploration constant, population size, or swarm size across seven algorithms, Covariance Matrix Adaptation Evolution Strategy (CMAES), Bayesian Optimization (BO), Exploitative Bayesian Optimization (EBO), Cross-Entropy (CE), Genetic Algorithm (GA), Gravitational Search Algorithm (GSA), and Particle Swarm Optimization (PSO). Each group represents an algorithm, with seven bars corresponding to parameters: Mean convergence Time (s), Peak Magnitude, Peak Timing, Peak Duration, normalized average of the root mean square of the muscles’ activations (EMG-RMS), Mean area under the convergence curve (AUC), and average rate of improvement (ARI). Error bars indicate the standard error of the mean absolute percent errors. EMG signals were recorded using seven surface electrodes placed on the user’s right leg, targeting the Vastus Medialis, Tibialis Anterior, Soleus, Rectus Femoris, Gluteus Maximus, Gastrocnemius Medialis, and Biceps Femoris muscles during treadmill walking.

The robustness of the seven global optimization algorithms was assessed through a ±10% hyperparameter perturbation test. The resulting mean absolute percent errors across seven performance metrics—Mean Convergence Time, Peak Magnitude, Peak Timing, Peak Duration, Normalized EMG-RMS, AUC, and ARI—are shown in Figure 9. The results indicate that CMA-ES, BO, EBO, CE, and GSA maintained relatively stable performance across metrics, exhibiting MAPE values below 25% for most parameters. In contrast, GA and PSO showed notably higher sensitivity, particularly in Peak Magnitude (≈59% for GA) and Peak Duration (≈56% for PSO). These results highlight that small hyperparameter perturbations affect population- and swarm-based optimizers more than gradient-free or covariance-adaptive methods.

Relative to our previous metabolic-based framework [2], the EMG-RMS objective produced different convergence profiles and sensitivities across optimizers. Notably, stable algorithms under metabolic landscapes [2] did not uniformly dominate under EMG, and the sensitivity analysis (Figure 9) revealed algorithm-specific vulnerabilities in Peak Magnitude and Peak Duration for GA and PSO. In contrast, CMA-ES, BO, EBO, CE, and GSA were less sensitive overall to ±10% hyperparameter perturbations. These findings indicate that optimizer choice should be objective-aware, particularly when the endpoint emphasizes muscle engagement rather than global energetics.

## 4. Discussion

The research question addressed in this study was: How do different surrogate models and optimization algorithms perform in simulating EMG-based HIL optimization for exoskeleton walking assistance? The objectives were to evaluate surrogate models for EMG-RMS prediction accuracy and to compare optimization algorithms based on key performance metrics, including convergence time and solution quality. We hypothesized that BO would outperform heuristic methods such as PSO in limited-evaluation scenarios due to its balanced exploration–exploitation strategy. Our findings partially supported this hypothesis, with GSA and PSO demonstrating unexpectedly strong performance across several metrics.

Among surrogate models, GB achieved the lowest relative absolute error (1.57%), outperforming PR (1.82%), SVR (1.65%), RF (1.65%), LR (2.14%), and kernel-based GP variants (1.65–1.73%) (Figure 3). These results suggest that ensemble learning methods like GB better capture the nonlinear characteristics of EMG-RMS cost landscapes than parametric alternatives [4]. Prediction plots (Figure 4) demonstrated strong agreement between predicted and actual EMG-RMS for GB and RF, with most discrepancies occurring in regions of high variability. Visualization of the EMG-RMS landscapes (Figure 5) confirmed that GB and RF generated smooth, well-structured surfaces with clearly defined optima, contributing to faster and more reliable optimization.

These findings extend prior metabolic landscape simulations [1,2] by incorporating EMG as a surrogate metric, which allows for shorter evaluation times and greater practicality in real-time HIL applications [4]. The robotic waist tether interface (Figure 1) integrates all key components, enabling closed-loop parameter tuning based on EMG feedback to estimate optimal assistance conditions. Compared to prior EMG-tuned controllers for leg swing tasks [4], our inclusion of multiple optimization algorithms highlights the effectiveness of GSA for navigating high-dimensional parameter spaces, in alignment with prior code structures and simulation frameworks [2].

Moreover, the application of ML techniques for EMG prediction from GRF and kinematics—such as those using ANNs [5] and response surface models [6]—offers a promising direction for enhancing surrogate model accuracy, especially in dynamic tasks where direct EMG recording is not feasible [7,8]. Musculoskeletal simulation approaches employing CMC have similarly predicted EMG-like activations from joint kinetics, providing phase-specific estimates of metabolic energy expenditure [11]. These results align with our perturbation-based data sources [21,22] and reinforce the potential of combining ML and physics-based models in future surrogate frameworks.

Our GB-based surrogate approach achieves competitive accuracy compared to prior EMG prediction models. For instance, a CNN–LSTM (Convolutional Neural Network—Long Short-Term Memory) architecture predicted lower-limb motion from EMG with an RAEP of 13.4% [24], while our GB surrogate achieved an RAEP of 1.57% in EMG-RMS. Other studies reported SVM-based EMG predictions with RAEP values around 2–3% for upper-limb tasks [25], highlighting GB’s superior performance. GP models for EMG forecasting during variable-speed gait demonstrated R^2^ > 0.85 [26], similar to our GP results (R^2^ > 0.99), yet ensemble models like GB still exhibited superior nonlinear mapping.

Simulation results revealed distinct performance profiles among the evaluated surrogate models and optimization algorithms. GSA achieved the best overall performance, with the shortest mean convergence time (~0.32 s) and the almost lowest EMG-RMS at the optimum (0.17 normalized units), as illustrated in Figure 6A,B. This aligns with GSA’s gravitation-inspired search mechanism, which is well-suited for exploring multimodal cost landscapes, such as those found in EMG-RMS simulations [1,2]. In contrast, PSO and CMAES produced higher EMG-RMS values (0.36 and 0.23, respectively), suggesting slower improvements (ARI values, 0.62 × 10^−5^ and 1.55 × 10^−5^, respectively) but more stable convergence (Figure 8C), likely due to their population-based search strategies that reduce susceptibility to local optima [1,2].

Our findings are consistent with previous efforts in the context of EMG-based robotic optimization. For example, a synergy-based HIL framework for hip exoskeletons improved synergy similarity scores to 0.80 [17], paralleling our GSA’s efficient optima identification (score 0.83). Similarly, LSTM-enabled EMG control for upper-arm exoskeletons achieved 0.96 prediction accuracy [27], supporting the use of ensemble models like GB for robust performance. EMG-driven adaptive impedance control for knee orthoses optimized torque application within 20 s [28], while our GSA achieved convergence in under 0.32 s. Other methods, such as nonlinear sliding mode control for elbow exoskeletons [29] and dynamic movement primitives (DMPs) with adaptive oscillators [30], also minimized EMG-based cost functions, which could lead us to future study to use those methods. These studies, along with reports of EMG-controlled shoulder and elbow exoskeletons achieving convergence within 5–10 s using fuzzy logic [31] and hybrid ANN–PSO systems yielding 15–20% metabolic cost reductions in lower-limb devices [32], collectively reinforce the competitive efficiency of our PSO and GSA implementations. GSA’s gravitational search framework further aligns with applications in exoskeleton stability optimization [33], suggesting potential extensions to multi-objective scenarios.

When compared to earlier surrogate-based optimization frameworks targeting metabolic cost [3], our EMG-centered approach demonstrated significantly faster convergence (GSA: <0.32 vs. 10–20 s in [1,2]), likely due to the lower variability and higher responsiveness of EMG signals compared to metabolic measurements. Our results also parallel findings from CMC-based simulations [11], where optimization of muscle parameters minimized joint moment errors—supporting the use of GB for precise EMG-RMS landscape modeling. The results suggest that pre-selecting GSA combined with a GB surrogate model offers an efficient solution for HIL optimization, substantially reducing the number of required experimental iterations and minimizing user fatigue.

Although compute time is generally less critical than human evaluation time in offline HIL optimization, it becomes highly relevant in scenarios requiring rapid, adaptive re-optimization [1,2]—such as Model Predictive Control (MPC)-like frameworks. In such use cases, where assistance parameters may need to adapt in real time to changes in speed, fatigue, or context, optimization algorithms must converge within sub-stride durations [1,2]. Our reported time-to-convergence values (Figure 8A; Table 5) offer insight into each algorithm’s suitability for these two regimes: slower but accurate methods (e.g., Bayesian variants) may be more appropriate for offline planning, while faster heuristics like GSA or PSO show potential for real-time or semi-online retuning. Metrics like AUC and ARI also provide valuable indicators of early-stage improvement, which is critical for minimizing user burden during HIL optimization [1,2].

In alignment with our prior simulation-based framework using metabolic cost as the optimization objective [2], the current EMG-RMS approach yielded comparable surrogate model performance, with GB achieving a RAEP of 1.57% compared to 0.66% for the metabolic model. GSA again emerged as the most effective optimizer, demonstrating both rapid convergence (<1 s) and strong solution quality (normalized EMG-RMS = 0.17), similar to its performance in minimizing metabolic cost (−1.06). However, while PSO was more efficient in the metabolic context (AUC = 0.24), GSA outperformed other methods in the EMG-based framework [2]. This divergence highlights EMG’s potential for faster, lower-burden HIL personalization in exoskeleton control compared to metabolic-based optimization.

The learned EMG-RMS landscape offers practical advantages for HIL exoskeleton optimization. By capturing the underlying relationship between assistance parameters and neuromuscular response, the surrogate model allows future sessions to begin in regions of the parameter space that are already known to be low-cost, significantly reducing the need for exhaustive re-exploration. This accelerates convergence and enhances user comfort. Furthermore, the EMG-based cost landscape serves as a foundational layer for future multi-objective optimization frameworks that could simultaneously incorporate other criteria such as metabolic cost, gait stability, or subjective comfort, enabling more holistic personalization of assistive strategies.

The newly introduced sensitivity analysis (Figure 9) provides critical insight into the robustness of the optimization algorithms. Overall, CMA-ES, BO, EBO, CE, and GSA demonstrated limited variation under ±10% hyperparameter changes, confirming their relative stability and reliability in EMG-based optimization tasks. Conversely, GA and PSO were more sensitive, particularly in Peak Magnitude and Peak Duration, where even small parameter perturbations caused large deviations. This behavior suggests that while population- and swarm-based methods can converge quickly, they require tighter hyperparameter control to maintain consistency. These findings support choosing covariance-adaptive and probabilistic methods (e.g., CMA-ES, BO) as robust candidates for personalized human-in-the-loop optimization, where model stability and reproducibility are critical.

EMG-RMS and metabolic cost [2] answer **complementary control questions;** the former prioritizes **muscle-level engagement and immediate responsiveness**, while the latter quantifies **whole-body economy** with slower dynamics. By establishing which surrogate–optimizer combinations are **stable and efficient** for an EMG objective, our results help pre-select **algorithms and hyperparameters** for **EMG-driven HIL** in patients with selective muscle weakness or limited endurance [34–38]. Equally important, these results enable **dual-objective designs** (e.g., minimizing metabolic cost while capping EMG-RMS in target muscles), where different optimizers may be preferred depending on **convergence speed** versus **final objective value**. In this sense, the present study **complements** our prior metabolic work. It provides an **objective-aware map** for algorithm selection that can **reduce human testing time**, **avoid redundant titration**, and **improve safety** before live HIL validation.

This study relied on simulated EMG-RMS landscapes, which may not fully capture inter-subject variability or real-time noise in human-in-the-loop experiments. Additionally, the framework lacks direct integration with metabolic cost measurements, which may limit the generalizability of the findings to broader physiological outcomes. The analysis was also constrained to a three-parameter optimization space (peak magnitude, peak timing, and duration), which may underestimate the complexity encountered in higher-dimensional or phase-specific control scenarios. Future work should expand the parameter space and investigate stability-related objectives, potentially integrating methods such as gait tube analysis or phase-dependent metrics to optimize both assistance effectiveness and dynamic stability [39–42]. This study was designed as a simulation-based investigation to evaluate surrogate-model and optimization-algorithm performance before experimental deployment. Although no physical exoskeleton trials were conducted, the surrogate models were trained on experimentally acquired EMG and biomechanical data, ensuring that the simulated responses represent realistic neuromuscular behavior. Similar simulation frameworks have been used successfully to predict HIL optimization trends in exoskeleton research [1,18,21,33,43,44]. Future work will integrate the most robust algorithms (CMA-ES and BO) into our waist-tether robotic platform to validate the predicted EMG-RMS reductions and convergence patterns in human subjects. This staged approach minimizes participant burden while ensuring experimental safety and reproducibility. The datasets [21,22] used in this study included ten healthy young adults with similar anthropometric characteristics. While this homogeneity ensured high signal consistency and reduced inter-subject variability—important for validating the surrogate-based optimization framework—it also limits generalizability to other populations. Future work will extend this simulation approach to **older adults and patients with motor impairments**, who often exhibit distinct neuromuscular activation patterns, stability strategies, and gait dynamics. Expanding the model to these populations will allow assessment of how algorithmic performance and sensitivity vary under clinical conditions, thereby improving the **translational relevance** of EMG-based HIL optimization for rehabilitation and assistive devices. This study used a robotic waist-tether system to simulate exoskeleton assistance by applying programmable forward traction forces near the body’s center of mass. While this system effectively captures the primary neuromechanical interaction between assistive force and muscle activation, it does not reproduce the full multi-joint torque distribution or bidirectional control characteristics of physical exoskeletons. The waist-tether approach was deliberately selected for its **simplicity, safety, and repeatability**, allowing algorithmic benchmarking without the confounding mechanical constraints of multi-joint devices. Prior studies using this system [21,22] have demonstrated that it elicits **neuromuscular adaptations similar to hip-exoskeleton assistance**, validating its use as a physiologically relevant surrogate for early-phase HIL optimization research. Future work will extend the present framework to **multi-joint robotic platforms** with active control at the hip, knee, and ankle, enabling direct assessment of posture guidance, bidirectional torque feedback, and energy exchange mechanisms in real exoskeleton hardware. The relative superiority of the optimization algorithms observed in this study is influenced by the nature of the EMG-based cost landscape and the algorithmic assumptions underlying each method. For instance, **GB** performs best when the surrogate model approximates smooth and structured nonlinear relationships with minimal noise, benefiting from its ensemble-based gradient correction. In contrast, **GSA** and **PSO** leverage population-based exploration and collective learning, which make them well-suited for **continuous, low-dimensional optimization problems** such as the three-parameter assistance space used in this study. However, their efficiency decreases in higher-dimensional or discontinuous cost landscapes, or when the response surface contains high stochasticity. These observations indicate that algorithmic performance is **not universally superior** but rather dependent on problem characteristics—particularly the smoothness and noise profile of the surrogate model and the sensitivity of the objective to small parameter perturbations. Furthermore, the promising results of **GSA** and **PSO** suggest their potential applicability to **real exoskeleton hardware experiments**, where adaptive exploration and convergence stability are essential. Future work will test these algorithms on physical waist-tether and hip-exoskeleton systems to assess their robustness in the presence of biological variability, sensor noise, and control latency, thereby linking simulation outcomes to practical human-in-the-loop optimization scenarios.

## 5. Conclusions

This study presented a comprehensive simulation framework for evaluating surrogate models and global optimization algorithms in EMG-based HIL optimization for robotic walking assistance. Among the nine machine learning models tested, GB emerged as the most accurate surrogate, effectively capturing nonlinear relationships within EMG-RMS landscapes. When integrated with GB, the GSA demonstrated the fastest and most consistent convergence performance across multiple evaluation metrics, including convergence time, AUC, and final EMG-RMS value. The combination of GSA and GB represents an efficient and robust configuration for simulating personalized exoskeleton control strategies, offering a promising alternative to time- and resource-intensive experimental tuning. By accurately estimating optimal assistance parameters based on simulated EMG responses, this approach enables the pre-selection of high-performing algorithm-model pairs, thereby reducing the number of required human trials and accelerating the development of individualized assistive solutions. These findings support the integration of machine learning-based surrogate modeling and algorithm benchmarking as a scalable method for advancing real-time HIL optimization in wearable robotics. Future work may expand this framework to include additional performance objectives, such as metabolic cost and gait stability, to support more holistic and adaptive control strategies in next-generation exoskeletons.

## Figures and Tables

**Table 1.** Comparison of related works on HIL optimization, EMG-based modeling, and exoskeleton personalization.

Study/Reference	Target Joint	Optimization Method	Objective Function	Personalization Type	Simulation or Real-Time	Key Contribution	Gap Addressed by Present Work
Desplenter & Trejos [19]	Elbow	None (model evaluation)	RMS torque error (1.67–2.19 Nm)	EMG-driven model	Simulation	Compared 7EMG activation models for motion estimation	No optimization; not hip-focused. Our work adds HIL optimization and hip-specific EMG modeling.
Slade et al. [20]	Ankle	Data-driven (logistic regression)	Metabolic energy (17–23% reduction)	Wearable sensors	Real-time	Real-time HIL optimization for ankle assistance	Real-time only, ankle-specific. Our work provides a multi-optimizer simulation for hip optimization.
Ma et al. [17]	Hip	HIL (Thompson sampling)	Muscle synergy (6.3% improvement)	sEMG feedback	Real-time	EMG-based synergy optimization for hip torque	Single optimizer; real-time only. Our work generalizes to multiple optimizers in simulation.
Li et al. [18]	Lower limbs	HIL (Thompson + collocation)	sEMG biofeedback	Hybrid (offline + online)	Hybrid	Combined trajectory and feedback optimization	Focused on multiple joints; not EMG-RMS optimization. Our work isolates hip neuromuscular control.
Kutulakos & Slade [1]	Ankle	Multiple (CMA-ES, PSO, GA)	Metabolic cost	Simulated HIL	Simulation	Compared optimizers for metabolic-based HIL	Ankle-focused and metabolic. Our work extends to hip with EMG-RMS surrogates.
Echeveste & Bhounsule [4]	Leg (swing)	Bayesian HIL	EMG for leg swing	Adaptive	Real-time	Demonstrated EMG-based fast convergence	Limited to Bayesian optimization and leg swing; not full gait. Our work extends to hip walking optimization.
Gonabadi et al. [2]	Hip	Seven global optimizers (GSA, PSO, CMA-ES, BO, CE, GA, EBO)	EMG-RMS (neuromuscular effort)	Simulated HIL with nine ML surrogates	Simulation	First multioptimizer, EMG-based surrogate framework for hip personalization	Bridges metabolic and neuromuscular optimization; reduces real-time burden; establishes algorithm sensitivity and reproducibility.

**Table 2.** Machine learning models and global optimization algorithms are used in the simulation framework. The listed models were employed to construct surrogate predictors of the average root mean square of the muscles’ activations (EMG-RMS). At the same time, the optimization algorithms were applied to identify low-cost assistance parameter combinations based on the surrogate predictions. Muscle activation signals were collected using seven surface EMG sensors placed on the user’s right leg during treadmill walking. Sensors were positioned over the Vastus Medialis, Tibialis Anterior, Soleus, Rectus Femoris, Gluteus Maximus, Gastrocnemius Medialis, and Biceps Femoris muscles. The model numbers are defined by “#”.

#	Machine Learning Models	Optimization Algorithms

1	Linear Ridge (LR)	Covariance Matrix Adaptation Evolution Strategy (CMAES)
2	Polynomial Ridge (PR)	Bayesian Optimization (BO)
3	Support Vector Regression (SVR)	Exploitative Bayesian Optimization (EBO)
4	Random Forest (RF)	Cross-Entropy Method (CE)
5	Gradient Boosting (GB)	Genetic Algorithm (GA)
6	Gaussian Process—Absolute Exponential (GPAE)	Gravitational Search Algorithm (GSA)
7	Gaussian Process—Matern 3/2 (GPM)	Particle Swarm Optimization (PSO)
8	Gaussian Process—Rational Quadratic (GPRQ)	-
9	Gaussian Process—Squared Exponential (GPSE)	-

**Table 3.** Summary of optimization algorithm configurations and initialization settings. All algorithms operated within normalized bounds [0, 1] for both Peak Magnitude, Peak Timings, and Peak Duration. The maximum number of function evaluations was set to 100 per run for all methods.

Algorithm	Population Size/Agents	Initialization Mean/Method	Key Parameters	Random Seed Used
CMA-ES	150	Mean: (0.5, 0.5, 0.5); σ = 0.3	λ = 15; Elite size = 3	rng(42)
Bayesian Optimization (BO)	N/A (sequential)	Uniform sampling in [0, 1]^3^	Acquisition: EI+; Exploration constant = 2.6	rng(42)
Exploitative BO (EBO)	N/A (sequential)	Uniform sampling in [0, 1]^3^	Acquisition: EI+; Exploration constant = 0.93	rng(42)
Cross-Entropy (CE)	150	Mean: (0.5, 0.5, 0.5); σ = 0.3	Elite fraction = 0.5; λ = 30 (2 × CMAES)	rng(42)
Genetic Algorithm (GA)	200	Uniform sampling in [0, 1]^3^	Crossover fraction = 0.8; Generations = ceil(200/20)	rng(42)
Gravitational Search Algorithm (GSA)	200	Uniform sampling in [0, 1]^3^	G_0_ = 100; α = 20; Max iterations = ceil (200/20)	rng(42)
Particle Swarm Optimization (PSO)	200	Uniform sampling in [0,1]^3^	Inertia-based updates; Swarm size = 20; Iterations = ceil (200/20)	rng(42)

**Table 4.** Performance summary of machine learning models used for EMG-RMS prediction. Each model was trained to construct a surrogate predictor of the average root mean square of the muscles’activations (EMG-RMS). The table reports nine machine learning models’ relative absolute error percentage (RAEP). EMG signals were recorded from seven surface sensors placed on the user’s right leg during treadmillwalking, targeting the VastusMedialis, Tibialis Anterior, Soleus, Rectus Femoris, Gluteus Maximus, GastrocnemiusMedialis, and Biceps Femoris muscles. The model numbers are defined by “#”.

#	Machine Learning Models	Relative Absolute Error of EMG-RMS (RAEP) (%)

1	Linear Ridge (LR)	2.14
2	Polynomial Ridge (PR)	1.82
3	Support Vector Regression (SVR)	1.65
4	Random Forest (RF)	1.65
5	Gradient Boosting (GB)	1.57
6	Gaussian Process—Absolute Exponential (GPAE)	1.66
7	Gaussian Process—Matern 3/2 (GPM)	1.66
8	Gaussian Process—Rational Quadratic (GPRQ)	1.65
9	Gaussian Process—Squared Exponential (GPSE)	1.73

**Table 5.** Summary of optimization results for seven algorithms integrated with the Gradient Boosting (GB) surrogate model. The table reports: (1) total number of evaluations to convergence, (2) mean convergence time (in seconds), (3) optimal assistance parameters—peak magnitude, peak timing, and peak duration—identified by each algorithm, (4) mean normalized EMG-RMS (average root mean square of the muscles’ activations) achieved at convergence, (5) mean area under the convergence curve (AUC) as a measure of optimization efficiency, and (6) average rate of improvement (ARI), indicating convergence speed. The evaluated algorithms include Covariance Matrix Adaptation Evolution Strategy (CMAES), Bayesian Optimization (BO), Exploitative Bayesian Optimization (EBO), Cross-Entropy (CE), Genetic Algorithm (GA), Gravitational Search Algorithm (GSA), and Particle Swarm Optimization (PSO). EMG signals were recorded from seven surface electrodes placed on the user’s right leg, targeting the Vastus Medialis, Tibialis Anterior, Soleus, Rectus Femoris, Gluteus Maximus, Gastrocnemius Medialis, and Biceps Femoris muscles during treadmill walking. **The model numbers are defined by “#”.**

#	Algorithm	Total Number of Evaluations	Mean Convergence Time (s)	Peak Magnitude	Peak Timing	Peak Duration	Normalized EMG-RMS	Mean AUC	Mean ARI

1	CMAES	100.00	0.33	0.01	0.73	0.52	0.23	0.13	1.55 × 10^−5^
2	BO	100.00	9.90	0.02	0.73	1.00	0.16	0.12	2.51 × 10^−5^
3	EBO	100.00	8.74	0.09	0.71	1.00	0.20	0.13	2.47 × 10^−5^
4	CE	100.00	0.33	0.09	0.93	0.17	0.22	0.13	2.17 × 10^−5^
5	GA	100.00	1.69	0.49	0.35	0.77	0.21	0.13	2.53 × 10^−5^
6	GSA	100.00	0.32	0.15	0.72	0.97	0.17	0.12	2.84 × 10^−5^
7	PSO	100.00	1.61	0.29	0.15	0.31	0.36	0.10	0.62 × 10^−5^

## Data Availability

The raw data supporting the conclusions of this article will be available from the authors on request.

## Abbreviations

The following abbreviations are used in this manuscript:

ANN: Artificial Neural Network
ARI: Average Rate of Improvement
AUC: Area Under the Curve
BO: Bayesian Optimization
CE: Cross-Entropy
CMAES: Covariance Matrix Adaptation Evolution Strategy
CMC: Computed Muscle Control
EBO: Exploitative Bayesian Optimization
EEG: Electroencephalogram
EMG: Electromyography
EMG-RMS: Average of The Root Mean Square of The Muscles’ Activations
ESN: Echo State Network
GA: Genetic Algorithm
GB: Gradient Boosting
GP: Gaussian Process
GPAE: Gaussian Process—Absolute Exponential
GPM: Gaussian Process—Matern 3/2
GPRQ: Gaussian Process—Rational Quadratic
GPSE: Gaussian Process—Squared Exponential
GRF: Ground Reaction Force
GSA: Gravitational Search Algorithm
HIL: Human-in-the-Loop
IMU: Inertial Measurement Unit
LR: Linear Ridge
ML: Machine Learning
PR: Polynomial Ridge
PSO: Particle Swarm Optimization
RF: Random Forest
RMS: Root Mean Square
SVR: Support Vector Regression
